# Unintentional Effects from Housing Enhancement Resulting in Functional Improvement in Spinal Cord–Injured Mice

**DOI:** 10.1089/neur.2022.0059

**Published:** 2023-01-27

**Authors:** Darlene A. Burke, Johnny R. Morehouse, Sujata Saraswat Ohri, David S.K. Magnuson

**Affiliations:** ^1^ Department of Neurological Surgery, University of Louisville School of Medicine, Health Sciences Campus, Louisville, Kentucky, USA.; ^2^ Kentucky Spinal Cord Injury Research Center, University of Louisville School of Medicine, Health Sciences Campus, Louisville, Kentucky, USA.

**Keywords:** BMS, climbing, enriched housing, locomotor recovery, spinal cord injury, TreadScan kinematics

## Abstract

It is well established that both positive and negative housing conditions of laboratory animals can affect behavioral, biochemical, and physiological responses. Housing enhancements have been shown to have beneficial effects on locomotor outcomes in rodents with spinal cord injury (SCI). Subsequent to an unplanned housing enhancement of the addition of a balcony to home cages by animal care personnel at a research facility, a retrospective analysis of multiple SCI studies was performed to determine whether outcomes differed before (four studies, *N* = 28) and after (four studies, *N* = 23) the addition of the balcony. Locomotor and morphological differences were compared after a mild-moderate T9 spinal cord contusion injury in wild-type mice. Post-injury assessments of locomotor function for 6 weeks included Basso Mouse Scale (BMS) and treadmill kinematic assessments (week 6). Balcony-housed mice showed greater improvements not only in basic locomotor functions (weight-supported stepping, balance) compared to those in standard housing, but also surpassed mice in standard housing without the balcony in higher-order locomotor recovery outcomes, including BMS late-stage recovery measures (paw, tail, and trunk indices). Additionally, balcony-housed mice had overall higher BMS scores, consistently attained more BMS subscores, and had better treadmill track width and stride length compared to those with no balcony. The housing enhancement of a balcony led to unforeseen consequences and unexpected higher recovery outcomes compared to mice in standard housing. This retrospective study highlights the importance of housing conditions in the key outcomes of locomotor recovery after incomplete contusive SCIs in mice.

## Introduction

A variety of rehabilitative methods have been used to promote functional locomotor recovery after spinal cord injury (SCI) in the rodent model, including activity, exercise, and step training with treadmill walking.^1–3^ Post-injury, great care is taken to ensure that essential medications, diet, housing conditions, and veterinary care are used for the well-being and recovery of laboratory animals. However, unanticipated events can result in unintentional effects on recovery outcomes.^4–6^

During a series of SCI research experiments at a research facility, the standard housing conditions of the laboratory animals were altered by the animal care facility, inadvertently changing the housing conditions of subsequent experiments compared to earlier identical studies. A balcony was added to the home cage of uninjured and mid-thoracic SCI mice to enhance well-being. After multiple experiments, it appeared that mice in the home cage with the balcony had better locomotor recovery post-injury compared to mice that had been housed in standard cages.

It is well established that the well-being of laboratory animals, as well as their behavioral, biochemical, and physiological responses, can be affected by housing conditions.^7,8^ Studies using environmental enrichment that include in-cage enhancements by inserting objects to encourage activities, such as tunneling, incline walking, and climbing behaviors, have shown improvements in locomotor recovery.^9,10^ Moreover, there is evidence that acute spontaneous effects referred to as “self-training” or “in-cage training” can have positive results on functional recovery.^11–13^ Housing enhancements as well as spontaneous self-training can unintentionally influence experimental research outcomes as a result of increased activity.^4,5^ Other environmentally related housing factors, including housing density, cage size, and design, can lead to experimental outcome variability and affect scientific validity.^4,6^ The following retrospective analysis was undertaken to determine whether an unintended housing enhancement during a series of SCI research experiments resulted in unexpected enhanced locomotor recovery in mild-moderate SCI in mice.

## Methods

### Design

A retrospective analysis was performed to compare four studies where mice were housed in standard cages (Table 1; studies A: *N* = 11, B: *N* = 5, C: *N* = 2, and D: *N* = 10; total = 28) with four studies where a balcony was added to the home cage (studies E: *N* = 8, F: *N* = 8, G: *N* = 3, and H: *N* = 4; total = 23). These eight experiments included four published^14^ and four yet-to-be published studies.

**Table 1.** Sample Sizes and Assessment Testing Time Points for Each of the Four Experiments Using Cages With and Without a Balcony

		**BMS**	**TreadScan**
**No balcony**	Sample size	Total (testing times)	Test week
Study A	11	7 (day 3, weeks 1–6)	Week 6
Study B	5	7 (day 3, weeks 1–6)	Week 6
Study C	2	7 (day 3, weeks 1–6)	Week 6
Study D	10	7 (day 3, weeks 1–6)	Week 6
Total	28		

**Balcony**	Sample size	Total (testing times)	Test week
Study E	8	7 (day 3, weeks 1–6)	Week 6
Study F	8	7 (day 3, weeks 1–6)	Week 6
Study G	3	6 (day 3, weeks 1, 3–6)	
Study H	4	7 (day 3, weeks 1–6)	
Total	23		
Grand Total	51		

Sample sizes are listed for each of the four experiments in which mice were housed in standard cages without a balcony and the four experiments with the balcony in the cage. Assessment testing weeks (post-injury) for BMS and TreadScan are shown for each study.

BMS, Basso Mouse Scale.

Identical personnel performed each of the surgery, animal care, breeding, behavioral assessments, morphological, and data handling throughout all eight experiments, ensuring that the consistency and continuity of all procedures and conditions were maintained throughout all eight experiments.

### Surgery

All studies consisted of wild-type (C57Bl/6) mice (Table 1). Mice received a 50-kdyn mild-moderate T9 contusion SCI (Infinite Horizon, Lexington, KY), as previously described.^15–17^

### Housing

**FIG. 1.**
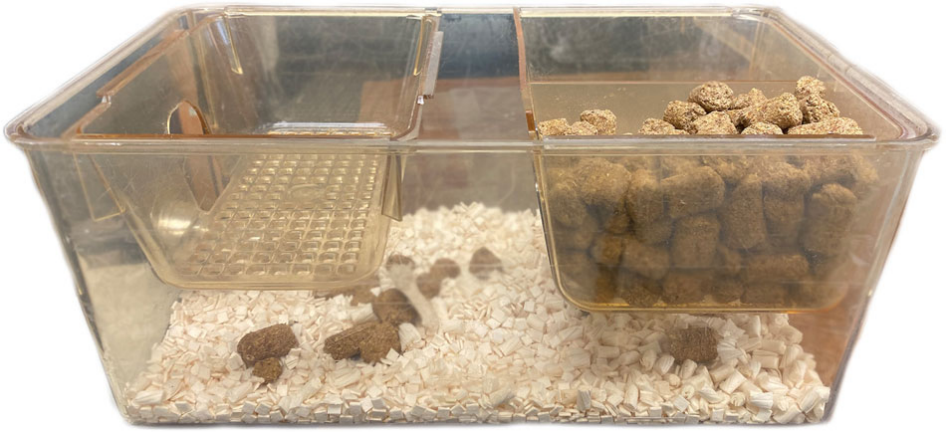
Side view of a standard mouse cage showing placement of the balcony at one side of the cage (left side) for the balcony group.

Mice were group housed in standard cages (3–5 per cage) for 6 weeks post-injury. Food and water were available *ad libitum*. In four experiments, a balcony was inserted in their home cages (7 in length × 5 in weight × 2¾ in depth) with easy accessibility for climbing (Fig. 1) through two side openings on opposite walls allowing passage to and from the cage floor. Animals were maintained on a 12-h light-dark cycle.

### Locomotor assessment

Hindlimb locomotor scores were assessed at six to seven time points using the Basso Mouse Scale (BMS; post-injury day 3, weekly for 6 weeks) by two trained observers blinded to experimental conditions.^18^ All components of BMS scoring were examined (Table 2, “Subscore”), plus three non-subscore components (Table 2, “Added”). Although the advantage of the BMS is its quick, convenient assessment of locomotor improvement in an open field setting and its parsing of the components (i.e., subscores) to examine the details of recovery, it is a non-linear categorical scale with a limited range of values (0–9). As recovery of function improves, the rise in the BMS score increases on an interval scale, regardless of the magnitude of the actual functional change. Relative to BMS changes that increase very slowly, subscore points add up more quickly as function increases. However, subscores are often highly variable, yielding non-significant differences. Combining variables can increase the effective range of values, decrease variability, and produce a measure that is more normally distributed.^19^ Thus, the BMS and BMS subscores were summed for each animal to yield a measure that more accurately indicates the magnitude of functional recovery.^19^

**Table 2.** Details of the Components Comprising the BMS Locomotor Assessment Scale

BMS	Scoring value and description		Subcomponents
Ankle Movement	0	No movement	Added
	1	Slight Movement (≤50%)	
	2	Extensive Movement (>50%)	
Plantar Placement	0	No plantar placement	Added
	1	Plantar Placement w/o Support	
	2	Plantar Placement w/ Support	
Dorsal Stepping	0	No Dorsal Stepping (0–5%)	Added
	1	Occasional Dorsal Stepping (>5 to ≤50%)	
	2	Frequent Dorsal Stepping (51–94%)	
	3	Consistent Dorsal Stepping (95–100%)	
Planter Stepping	0	No Plantar Stepping (0–5%)	Subscore
	1	Occasional Plantar Stepping (>5 to ≤50%)	
	2	Frequent Plantar Stepping (51–94%)	
	3	Consistent PlantarStepping (95–100%)	
Coordination	0	No Coordination	Subscore
	1	Some Coordination (≤50%)	
	2	Mostly Coordinated (>50%)	
Paw Rotation	0	Rotated at Initial contact & Rotated at Lift Off	Subscore
	1	Parallel at Initial contact & Rotated at Lift Off	
	2	Parallel at Initial contact & Parallel at Lift Off	
Trunk Score	0	Severe Trunk Instability	Subscore
	1	Mid Trunk Instability	
	2	Normal Trunk Instability	
Trunk Events	Number of events		Added
Tail	0	Tail Down or Tail Up & Down	Subscore
	1	Tail Up	

To the right are the locomotor properties that are represented in the subscore when a subscore of 5.5 on the left and right sides (frequent-consistent plantar stepping, no coordination) is attained. The remaining BMS outcome component properties that are not part of the subscore were scored in the same manner.

BMS, Basso Mouse Scale.

### Kinematic treadmill walking

Treadmill walking was assessed (Single Lane Gait Analysis Treadmill; Columbus Instruments; Columbus, OH), as previously described.^20^ Briefly, a high-speed digital video camera recorded the ventral view of the treadmill belt as animals walked across. Images were recorded at 100 frames/s and analyzed using MaxTRAQ (Innovision Systems Inc; Columbiaville, MI). Acceptable passes included walking in the middle of the treadmill, stepping with few lateral deviations, and stepping without pauses.

### Spared white matter

At 6 weeks post-SCI, mice were perfused and spinal cords processed, sectioned, and mounted, as previously described.^16,17^ To detect the extent of spared white matter (SWM) at the injury epicenter, iron eriochrome cyanine with an alkali differentiator was used to stain myelin, as previously described.^15,21,22^ Sections were imaged, quantified, and the epicenter identified, as previously described.^16,17^ SWM from the experimental group was normalized to average white matter content at identical spinal levels from a separate cohort of naïve control animals.

### Statistical analysis

Four BMS outliers (≥2.5 standard deviations [SDs]) were removed. BMS scores and component left- and right-side values were not different and were averaged. Time course of recovery was assessed by averaging acute (days 3 and 7), chronic (weeks 5 and 6), and all (D3, weeks 1–6) time points. Group comparisons of BMS component scores were performed using non-parametric Mann-Whitney U tests. Frequency counts were compared using binomial proportion tests, corrected for small sample size when appropriate.^23^ TreadScan assessments were normalized to baseline (BL) values (week 6/BL × 100) and analyzed using repeated-measures analysis of variance (ANOVA; Group and Side main effects and interaction; Track Width: Group and Front/Rear main effects and interaction), followed by Bonferroni *post hoc t*-tests. Independent *t*-tests (between means with equal or unequal variances, as appropriate) were limited to reduce the probability of a type 1 error and were used to compare the balcony groups' kinematic speed and coordination measures.^24^ Non-parametric Spearman rank correlations were performed among BMS, TreadScan, and SWM outcomes.^23^ Values represent mean ± SD. Data were analyzed using SPSS statistical software (v27; SPSS, Inc., Chicago, IL).

## Results

### Basso Mouse Scale, Basso Mouse Scale subcomponents

Balcony group mice had higher BMS (all and chronic), BMS + subscore (all, Fig. 3A and chronic), and plantar stepping (all and chronic), plus fewer negative trunk events (all), compared to those in the no-balcony group (Table 3, upper panel). At acute post-injury time points, balcony-housed mice had better plantar paw placement compared to those housed without a balcony (Table 3, upper panel).

**Table 3.** Results of the Tests Comparing the Balcony and No Balcony Groups

	All	Acute (D3 & 7)	Chronic (W5 & 6)
BMS	M-W U U = 9943,* p* < 0.001	M-W U	M-W U U = 651,* p* = 0.001
Plantar Placement		U = 221, *p* < 0.05	
Plantar Stepping	U = 6065, *p* < 0.001		U = 671, *p* < 0.005
Trunk Events	U = 11721, *p* = 0.05		
BMS+Subscore	U = 10003, *p* < 0.001		U = 604, *p* = 0.001

	ANOVA Group main effect	Bonferroni* post hoc *t-test	
Stride Length Forelimb	F = 8.6, df = 1,50, *p* = 0.005	Rt: *p* < 0.05; Lt: *p* = 0.073	
Hindlimb	F = 11.9, df = 1,50, *p* = 0.001	Rt: *p* < 0.05; Lt: *p* < 0.05	
Foot Base of Support	F = 14.3, df = 1,49, *p* < 0.001	Rt: *p* < 0.05; Lt: *p* < 0.05	
Front Track Width	F = 5.4, df = 1,46, *p* < 0.05	*p* < 0.05	
Average Speed	t = 0.73, df = 25, *p* > 0.05		
Regularity Index	t = 0.19, df = 25, *p* > 0.05		
Central Pattern Index	t = 0.68, df = 25, *p* > 0.05		
Plantar Stepping Index	t = 0.10, df = 25, *p* > 0.05		

Upper panel shows the significant results of the M-W U tests comparing the balcony and no balcony groups' ranked BMS scores and their components, and the lower panel shows the results of the mean group comparisons (repeated-measures ANOVA, independent *t*-tests) of the kinematic outcome measures.

D, day; W, week; Rt, right side; M-W U, Mann-Whitney U; BMS, Basso Mouse Scale; ANOVA, analysis of variance.

**FIG. 2.**
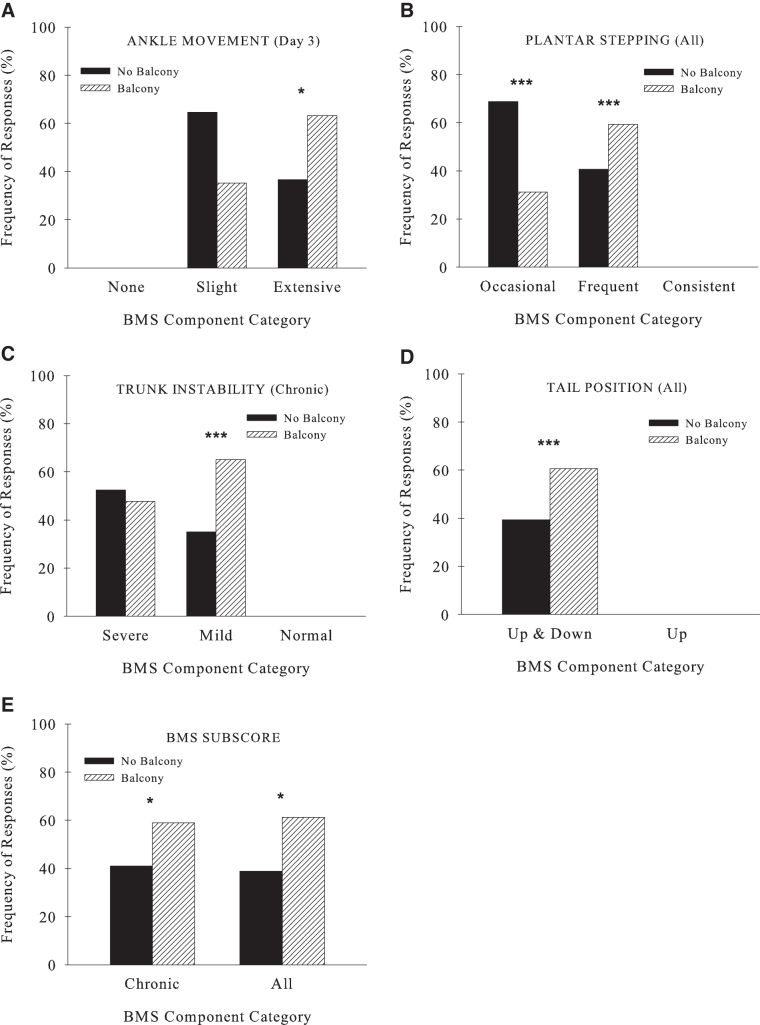
Frequency comparisons of BMS components between the balcony and no balcony groups are shown for locomotor function outcomes of the BMS (**A**) ankle movement, (**B**) plantar stepping, (**C**) trunk stability, (**D**) tail position, and (**E**) subscore. *p < 0.05, ***p ≤ 0.005. BMS, Basso Mouse Scale.

These results were supported by frequency counts within the levels of the BMS components that describe specific details of functional recovery (Table 4). Balcony group mice had more frequent extensive ankle movement compared to standard housed mice (Fig. 2A). Mice housed with a balcony more often achieved frequent stepping (chronic and all, Table 4). Conversely, most of the mice housed without a balcony were capable of only occasional stepping (chronic and all, Fig. 2B) and had poor trunk stability (chronic, Fig. 2C) and tail position (all, Fig. 2D).

**Table 4.** Comparison of Frequency Counts (Percent Total Balcony and No Balcony) Between the Balcony and No Balcony Groups for Weight Support, BMS Components, and Subscores

BMS			Frequency count		Binomial pioportion	
Component	Timepoint	Description	No balcony	Balcony	z	*p* value
**Ankle Movement**	Day 3	None	2/4 (50%)	2/4 (50%)	N/A	
		Slight	11/17 (65%)	6/17 (35%)	1.8	n.s.
		Extensive	11/30 (37%)	19/30 (63%)	2.1	*p* < 0.05
**Weight Support**	Acute		49/53 (92%)	38/42 (90%)	N/A	
	Chronic		48/49 (98%)	42/42 (100%)	N/A	
**Plantar Stepping**	Acute	Occasional	42/75 (56%)	33/75 (44%)	1.5	n.s.
		Frequent	6/16 (38%)	10/16 (63%)	1.5	n.s.
		Consistent	0	0		
	Chronic	Occas.	37/47 (79%)	10/47 (21%)	6.8	*p* < 0.001
		Freq.	55/127 (43%)	72/127 (57%)	2.2	*p* < 0.05
		Consis.	2/4 (50%)	2/4 (50%)	N/A	
	All	Occasional	179/260 (69%)	81/260 (31%)	9.3	*p* < 0.001
		Frequent	112/275 (41%)	163/275 (59%)	4.4	*p* < 0.001
		Consistent	2/5 (40%)	3/5 (60%)	N/A	
**Trunk Instability Score**	Acute	Severe	1/2 (50%)	1/2 (50%)	N/A	
		Mild	2/6 (33%)	4/6 (67%)	N/A	
		Normal	0	0		
	Chronic	Severe	11/21 (52%)	10/21 (48%)	N/A	
		Mild	14/40 (35%)	26/40 (65%)	2.8	*p* = 0.005
		Normal	0	0		
	All	Severe	15/35 (43%)	20/35 (57%)		n.s.
		Mild	37/99 (37%)	62/99 (63%)	3.7	*p* < 0.001
		Normal	0	0		
**Tail Position**	Chronic	Down/Up&Dn	25/60 (42%)	35/60 (58%)	1.9	n.s. (*p* = 0.064)
		Up	N/A	N/A		
	All	Down/Up&Dn	52/132 (39%)	80/132 (61%)	3.5	*p* < 0.001
		Up	0/2	2/2	N/A	
**Trunk Events**	Acute		9/22 (41%)	13/22 (59%)	1.2	n.s.
	Chronic		26/47 (55%)	21/47 (45%)	1	n.s.
	All		64/126 (51%)	62/126 (49%)	N/A	
**Subscore**	Acute		3/8 (38%)	5/8 (63%)	1.0	n.s.
	Chronic		25/61 (41%)	36/61 (59%)	2.0	*p* < 0.05
	All		52/134 (39%)	82/134 (61%)	2.0	*p* < 0.05
**Coordination**	Chronic	None	23/55 (42%)	32/55 (58%)	1.7	n.s.
		Some	2/6 (33%)	4/6 (67%)	N/A	
		Most	0	0		
	All	None	44/119 (37%)	75/119 (63%)	4.2	*p* < 0.001
		Some	8/15 (53%)	7/15 (47%)		n.s.
		Most	0	0		
**Paw Rotation**	Acute	2 Rotated	4/6 (67%)	2/6 (33%)	N/A	
		1 Rot./1 Par.	2/10 (20%)	8/10 (80%)	3.4	*p* = 0.001
		2 Parallel	0	0		
	Chronic	2 Rotat.	13/30 (43%)	17/30 (57%)	1.0	n.s.
		1 Rot./1 Par.	36/86 (42%)	50/86 (58%)	2.2	*p* < 0.05
		2 Parall.	1/6 (17%)	5/6 (83%)	2.8	*p* = 0.005
	All	2 Rotated	22/72 (31%)	50/72 (69%)	5.1	*p* < 0.001
		1 Rot/1 Par.	80/188 (43%)	108/188 (57%)	2.9	*p* < 0.005
		2 Parallel	2/8 (25%)	6/8 (75%)	2.3	*p* < 0.05

N/A indicates a sample size too small and/or values approximating 50%.

BMS, Basso Mouse Scale; n.s., not significant.

**FIG. 3.**
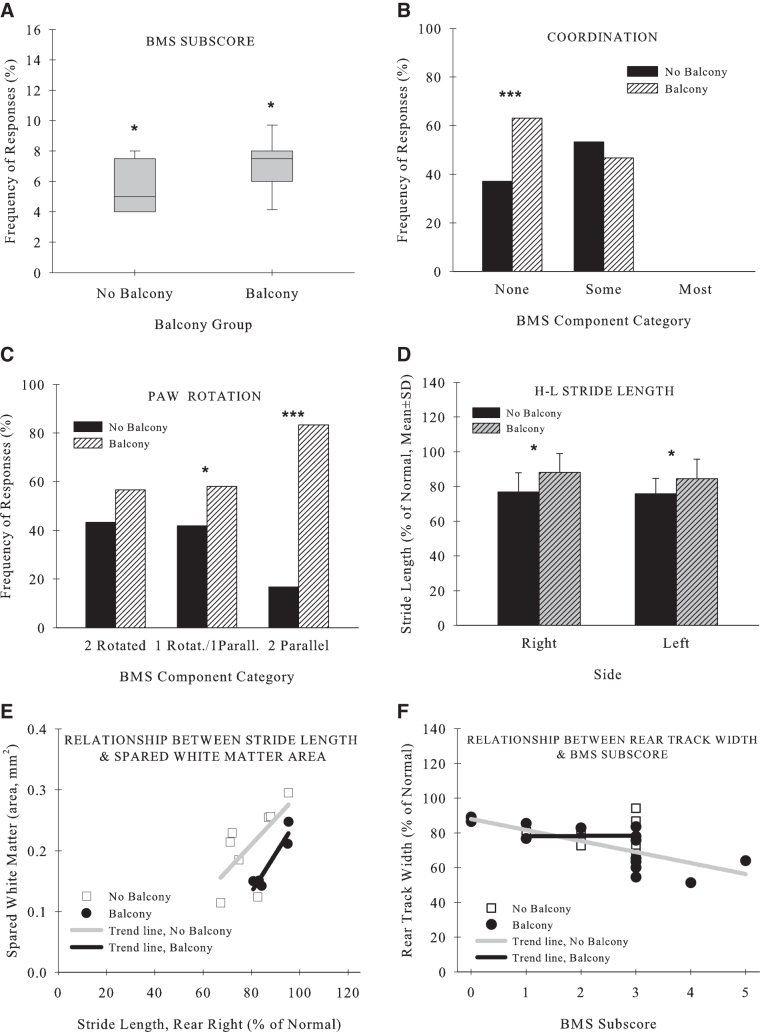
Results of the balcony and no-balcony group comparisons are shown for locomotor function outcomes of (**A**) BMS subscore (box-plot values = median ±10th and 90th percentiles), (**B**) forelimb-hindlimb coordination, (**C**) paw rotation, and (**D**) kinematic hindlimb rear right and left stride length. Non-parametric ranked correlations show the significant relationships between (**E**) stride length and SWM in the no-balcony group and (**F**) rear track width and BMS subscores in the balcony group. *p < 0.05, ***p ≤ 0.005. BMS, Basso Mouse Scale; SWM, spared white matter.

More mice in the balcony group received a subscore (BMS, ≥5) compared to the group without a balcony (all and chronic; Fig. 2E, Table 4), thus indicating greater locomotor improvement. Although ∼50% of animals in both groups had some coordinated walking (Fig. 3B), unexpectedly, there was a significantly higher number of balcony-housed mice with no coordination compared to those without a balcony (all). This likely resulted because there were more mice in the balcony group initially who were able to walk and therefore this produced more instances of non-coordinated stepping. This is supported by the more frequent stepping in the balcony group compared to mice housed without the balcony (Fig. 2B). Mice housed with the balcony also had better paw positions (1–2 parallel: acute, chronic, and all; chronic: Fig. 3C; both parallel: chronic and all, Table 4).

### Kinematic treadmill

Mice with the balcony in the cage had more normal stride lengths (normal = 100%) when walking on the treadmill compared to those without a balcony (forelimb: right; hindlimb: right and left; Fig. 3D, Table 3, lower panel). Conversely, mice without the balcony had shorter stride lengths, indicating that they took shorter, choppier steps. Poorer stride lengths correlated with the lower SWM area values in the no-balcony group (*r*_s_ = 0.79, *p* < 0.05, *N* = 8; Fig. 3E). Two measures related to stability and balance—foot base of support and front track width—were better in the balcony versus no-balcony group (Table 3, lower panel). Mice housed with the balcony also exhibited better trunk stability, resulting in smaller changes in their rear track width. This was supported by the increasing BMS subscores in the balcony group, with a corresponding decrease in rear track width (*r*_s_ = −0.78, *p* < 0.001, *N* = 14; Fig. 3F).

Consistent with the observation of no significant differences in BMS coordination, no differences were found in the indices of coordinated stepping. The Coordinated Pattern Index and the Regularity Index (measures of correct stepping patterns with or without dorsal steps), as well as the Plantar Stepping Index (a measure of forelimb to hindlimb stepping), did not differ between the balcony groups (all, Table 3, lower panel). In addition, stepping speed on the treadmill revealed no significant difference (all, Table 3, lower panel).

### Spared white matter

Terminal assessment of the amount of SWM 6 weeks post-injury yielded no significant group difference (balcony: 0.18 ± 0.05, *N* = 5; no balcony: 0.20 ± 0.07, *N* = 9; *t* = 0.55, *df* = 12, *p* > 0.05).

## Discussion

A retrospective study of multiple studies after a housing enhancement by animal care personnel in a research laboratory demonstrates that substantial functional locomotor improvements can occur unintentionally from enhanced housing. Studies have shown that a variety of techniques to enhance locomotor improvement can occur after not only formal training and exercise regimens, but also spontaneously and from in-cage enhancements that promote activity.^2,6,12,13,25^ However, it is unclear what relevant factors contribute to the recovery in each of these techniques. This becomes further complicated by recovery behaviors that are task specific.

A key component necessary to improve functional locomotor recovery and enhance plasticity begins with weight-supported stepping.^26–28^ Although body weight support (BWS) is a fundamental component of functional recovery, it is likely that rehabilitative training incorporating BWS alone is not sufficient to restore locomotor function beyond what occurs “spontaneously.”^3^ Injured animals undergoing rehabilitative activities, such as step training with BWS, either through a harness and treadmill or in shallow water, have the ability to produce well-organized patterns of stepping on a treadmill. However, they are unable to do so overground because of a lack of balance.^29–31^

Locomotor training techniques have revealed the need not only for load-bearing weight support for functional improvement, but also the importance of proprioceptive input.^3,32–34^ The modulation of muscle output by sensory inputs leads to “fine-tuning” in which the basic locomotor pattern generated by the central pattern generator is fine-tuned to adapt to terrain and walking surface changes.^35,36^ This fine-tuning is dependent on the step phase (stance vs. swing) and thus is important for both intra- and interlimb coordination.^33,37^

The outcome measures of the BMS, its components, and kinematic assessments utilized to assess locomotor functional recovery in the present studies can be categorized according to basic (e.g., weight support and plantar stepping) and higher-order (e.g., paw and tail positions, trunk stability and coordination) functional outcomes. In the present studies, balcony group mice showed greater improvement in the BMS's early stages of recovery for basic ankle limb movements, plantar placement, and plantar stepping, in addition to kinematic foot base of support. Thus, mice with the balcony improved more in all basic locomotor recovery abilities compared to mice without a balcony, first developing weight support, then plantar placement of the paw, followed by weight-supported plantar stepping.

As previously alluded to, the relationship between trunk control and balance is an integral feature in the successful acquisition of basic locomotor function after SCI. Moreover, it also plays an important role in the subsequent improvement of more complex higher-order locomotor abilities. Mice housed with the balcony performed better in multiple balance-related outcome measures, including paw rotation, trunk stability, and tail position, with a corresponding reduction in adverse trunk events. Also, kinematic outcomes for stride length and track width were better for balcony-housed mice compared to those mice housed in standard cages. These achievements illustrate that mice housed with access to the balcony advanced beyond basic weight-supported stepping, exhibiting continued improvements in stability and balance over and above those of standard housed mice.

The progression to fine-tuned complex motor behaviors representing higher-order recovery also was better in balcony-housed mice compared to those housed without the balcony. This is exemplified by balcony group mice having higher overall BMS and BMS + subscore results, clearly demonstrating better overall locomotor performance. In addition, other evidence of higher-order functional outcome measures supporting greater improvements in mice housed with a balcony compared to those in standard housing include the significant relationship among locomotor and kinematic functional outcomes. Specifically, there was a positive relationship between BMS subscores and kinematic rear track width for balcony-housed mice only, suggesting that the continued improvement in stepping and finer details of locomotion were related to recovery of balance.

However, neither BMS nor TreadScan outcome measures for coordination revealed significant differences between the balcony and no-balcony groups. It is suggested that climbing up onto the balcony and other activities promoted by the presence of the balcony likely contributed to the ability for plantar placement of the hindlimbs. In turn, it is conceivable that an increase in basic plantar placement was associated with increased muscle strength attributable to repeated limb-loading at acute time points with or without improvement in balance.

Another important indicator supporting functional improvement can be gleaned from the morphological assessment of SWM. In the current studies, direct comparisons of the SWM area between groups revealed no significant differences; however, the relationship between SWM and stride length were different for the two groups. Animals without the balcony had poor stride lengths while walking on the treadmill, and poor stride lengths were significantly related to poorer white matter sparing outcomes. Taken together, these assessments, along with the morphological findings, confirm the superiority of fine-tuned locomotor skills for mice with access to the balcony.

Adkins and colleagues promoted the concept that different skills result in different physiological changes and that the properties of strength, endurance, and skill training are necessary components to accomplish locomotor improvement.^1^ A common goal of rehabilitative methods that utilize training is the development of a skill that is retained post-training. It could be argued that the addition of the balcony resulted in increased exercise through the activity of climbing and constituted the attainment and practice of an acquired skill without formal or applied training. Although activity levels were not directly measured in the current studies, climbing is a natural behavior in mice and, given the opportunity, they will spend a considerable amount of time engaging in high-energy activities, such as climbing and wheel running, compared to walking or other natural (grooming, eating, and sleeping) behaviors.^38,39^ As concluded by Büttner, “climbing is obviously a regular component of activity…and is a factor in locomotor activity.”^39^

In the present studies, where level and severity of the SCI did eliminate the ability to climb acutely post-injury in all animals, it is likely that balcony-housed mice had increased exercise by engaging in climbing. This is supported by a study using rats with mild-moderate SCI housed in environmentally enriched cages with activities for climbing that reported significant improvement in locomotor scores and subscores, as well as the kinematic outcome measures, thus attaining higher-order fine-motor skills as compared to animals housed without enrichment.^40^ Thus, given the opportunity, animals with the capability will engage in both their “natural” (climbing) and “un-natural” (wheel-running) behaviors if present and available in their environment.^41^

A higher percentage of balcony-housed mice were able to achieve frequent stepping compared to those without the balcony (59 vs. 42%, respectively), suggesting that the activities made possible by the balcony (i.e., climbing and other behaviors) influenced the capacity to step. Thus, the specific preference for climbing compared to other activities demonstrated previously support the contention that mice in the present studies were highly likely to engage in the activity of climbing, even though this behavior was not monitored.^38,39^

The improved outcomes reported here suggest that the activity of climbing positively influenced at least two of the three training-related tasks as outlined by Adkins and colleagues: skill and strength, and possibly the third, endurance for locomotor recovery.^1^ Moreover, climbing likely involves most of the five levels of motor control needed for locomotor recovery as defined by Prochazka and Yakovenko: skeletomuscular and stretch reflex properties associated with load compensation, body motion and pattern generation, adaptation and prediction, and behavioral goals and context.^33^

The present studies show a clear improvement in locomotor recovery in SCI mice after exposure to a housing enhancement (a balcony), adopted primarily to improve animal well-being and reduce stress. The incorporation of a balcony in the present studies represents both a type of environmental enrichment with increased activity levels and also, simultaneously, the opportunity for in-cage self-training that involves whole-body movements, trunk control, limb strength, and balance. Results support the contention that the addition of a balcony provided training opportunities that allowed critical milestones in basic and fine-motor skills to be acquired that surpassed those of animals in standard cages without a balcony. These studies reveal that substantial functional locomotor improvements can occur unintentionally from enhanced housing that encourages an increase in general activity and specific skill-learning, such as climbing.

## Conclusion

The capability of experimental studies to discern effects from experimental manipulations versus extraneous, unintended influences is imperative for scientific validity. In the current body of studies, the unplanned addition of a balcony to standard cage housing resulted in unintended effects to experimental outcomes in SCI mice. Results demonstrated broad functional improvement, encompassing both basic and higher-order locomotor abilities in mice housed with the balcony compared to no balcony. The present results clearly illustrate that significant functional changes in locomotor outcomes can occur unintentionally after mild-moderate contusion SCI from enhanced housing that encourages an increase in general and specific activity such as climbing.

## Abbreviations Used

ANOVA: analysis of variance
BL: baseline
BMS: Basso Mouse Scale
BWS: body weight support
SCI: spinal cord injury
SD: standard deviation
SWM: spared white matter
